# Dietary patterns of Brazilian adults in 2008–2009 and 2017–2018

**DOI:** 10.11606/s1518-8787.2021055003437

**Published:** 2021-11-12

**Authors:** Anna Beatriz Souza Antunes, Diana Barbosa Cunha, Valéria Troncoso Baltar, Josiane Steluti, Rosangela Alves Pereira, Edna Massae Yokoo, Rosely Sichieri, Dirce Maria Marchioni

**Affiliations:** I Universidade do Estado do Rio de Janeiro Instituto de Medicina Social Programa de Pós-Graduação em Saúde Coletiva Rio de Janeiro RJ Brasil Universidade do Estado do Rio de Janeiro. Instituto de Medicina Social. Programa de Pós-Graduação em Saúde Coletiva. Rio de Janeiro, RJ, Brasil; II Universidade do Estado do Rio de Janeiro Instituto de Medicina Social Departamento de Epidemiologia Rio de Janeiro RJ Brasil Universidade do Estado do Rio de Janeiro. Instituto de Medicina Social. Departamento de Epidemiologia. Rio de Janeiro, RJ, Brasil; III Universidade Federal Fluminense Instituto de Saúde Coletiva Departamento de Epidemiologia e Bioestatística Niterói RJ Brasil Universidade Federal Fluminense. Instituto de Saúde Coletiva. Departamento de Epidemiologia e Bioestatística. Niterói, RJ, Brasil; IV Universidade Federal de São Paulo Instituto de Saúde e Sociedade Departamento de Políticas Públicas e Saúde Coletiva Santos SP Brasil Universidade Federal de São Paulo. Instituto de Saúde e Sociedade. Departamento de Políticas Públicas e Saúde Coletiva. Santos, SP, Brasil; V Universidade Federal do Rio de Janeiro Instituto de Nutrição Josué de Castro Departamento de Nutrição Social e Aplicada Rio de Janeiro RJ Brasil Universidade Federal do Rio de Janeiro. Instituto de Nutrição Josué de Castro. Departamento de Nutrição Social e Aplicada. Rio de Janeiro, RJ, Brasil; VI Universidade de São Paulo Faculdade de Saúde Pública Departamento de Nutrição São Paulo SP Brasil Universidade de São Paulo. Faculdade de Saúde Pública. Departamento de Nutrição. São Paulo, SP, Brasil

**Keywords:** Adult, Feeding Behavior, Factor Analysis, Socioeconomic Factors, Diet Surveys

## Abstract

**OBJECTIVES:**

To identify dietary patterns among Brazilian adults based on the National Dietary Surveys (INA – Inquéritos Nacionais de Alimentação) in 2008–2009 and 2017–2018, and to verify in the second period the adherence to the patterns according to sociodemographic factors and Brazilian regions.

**METHODS:**

We analyzed the first of two days of adults’ food consumption (19–59 years) in INA data from 2008–2009 (n = 21,630) and 2017–2018 (n = 28,901). Dietary patterns were derived by exploratory factor analysis from 19 food groups, considering the complexity of the sample design. We evaluated the factor scores according to sex, age group, region, per capita income, and education for the INA data in 2017–2018.

**RESULTS:**

We identified three patterns in the two surveys: (1) “traditional”, characterized by rice, beans, and meat; (2) “breads and butter/margarine”, characterized by breads, oils, and fats (including margarine/butter) and, coffee and teas in 2008–2009; and (3) “western”, characterized by sodas, pizzas, snacks, flour, pasta, and sweets in 2017–2018. The “traditional” pattern had greater adherence among men, residents of the Midwest region and individuals with incomplete primary education. “Bread and butter/margarine” pattern had greater adherence among males, individuals aged between 40 and 59 years, from the Southeast region, and with income between 1 and 2 minimum wages per capita. Male individuals, aged between 19 and 39 years, from the South region, with per capita income greater than two minimum wages, and education level equal to or greater than primary education showed greater adherence to the “western” pattern.

**CONCLUSION:**

The dietary patterns identified in 2008–2009 and 2017–2018 were similar, and we observed the maintenance of the “traditional” pattern, which includes rice, beans, and meat. Adherence to the dietary patterns varies according to sex, age group, region, per capita income, and education level.

## INTRODUCTION

It is difficult to elucidate the role of diet in promoting health and preventing disease given the complexity of the interaction between food and nutrients. In addition to total energy intake, diet is probably the most important discriminating factor within and between populations. Dietary composition is reflected in dietary patterns, explaining why this type of analysis has its popularity growing^1^.

In Brazil, dietary patterns have been investigated in several studies in recent decades, and the characteristic combination of the Brazilian diet, based on rice and beans, a dietary pattens generally called “traditional”^2^. Unlike the isolated analysis of foods and nutrients, the analysis of dietary patterns considers the relationships between the different food items consumed, encompassing different combinations^1^. Estimates in dietary patterns upon detecting everyday practices support the formulation of dietary guidelines, food guides, programs, and public policies.

However, data on the evolution of dietary patterns in the country are still scarce^8^, despite changes in the Brazilian diet over the years. Among these changes, we highlight the important increase in the participation of ultra-processed foods, the reduction in the consumption of beans, and the increase in the consumption of fruits and vegetables (still inadequate)^9^.

The National Dietary Survey (INA - *Inquérito Nacional de Alimentação* ), conducted in a sub-sample of the Household Budget Survey (POF - *Pesquisa de Orçamentos Familiares* ) since the 2008–2009 edition, render estimates of individual food intake for the entire Brazilian population^10 , 11^. This study shall identify the dietary patterns among Brazilian adults based on data from INA 2008–2009 and 2017–2018, and to verify the adherence to the patterns according to sociodemographic factors and Brazilian regions in the second period.

## METHODS

### Study Population

The population participating in the INA 2008–2009 and 2017–2018 editions, a module of the POF, was studied. A nationwide survey carried out by household sampling. POF collects information on family expenses, living conditions, and consumption habits of Brazilian families. Details about the sampling process and the definition of the master sample can be obtained from official publications of the Brazilian Institute of Geography and Statistics (IBGE - *Instituto Brasileiro de Geografia e Estatística* )^10 , 11^.

For the INA 2008–2009, were collected data of the personal food intake for all residents aged 10 years and over residing in 13,569 randomly selected households. These households correspond to a sub-sample of 24.3% of the 55,970 households investigated in the POF, totaling information on 34,003 residents. For the 2017–2018 edition, individual food intake information for the same age group (≥ 10 years) was collected in 20,112 randomly selected households, corresponding to a sub-sample of 34.7% of the 57,920 households investigated in the POF, totaling information from 46,164 residents.

The present analysis includes adult residents (aged 19 to 59), for both sex. Pregnant and lactating women during the collection period (1,111 in 2008–2009 and 1,249 in 2017–2018) were excluded. The final sample made up a total of 21,630 people in 2008–2009, and 28,901 in 2017–2018.

### Individual Food Consumption

We obtained the individual food consumption data from the personal food consumption questionnaire (POF7 block) in both editions of the surveys studied. The instrument used to collect information about food and beverages consumed indoors or outdoors was different in the two editions.

In INA 2008–2009, each resident recorded his individual intake on two non-consecutive days. The individuals were instructed to note and report in detail the name of the food consumed, the type of preparation, the measure used, the amount consumed, and the time and place of consumption (indoor or outdoor). To collect food record (FR), a research agent reviewed records with the residents and inserted the information to a laptop computer, using a data entry program created for this purpose. The software contained a food and beverage database of approximately 1,500 registered items.

In INA 2017–2018, food consumption was assessed by two 24-hour dietary recalls (R24h) at the respondent’s home, on non-consecutive days. The interview was developed following a structured script in sequential stages, based on the Multiple-Pass Method^12^. Repeating the previous edition, research agents also used a data entry program, installed on a tablet. The program provided information on the amount consumed (unit of measure and quantity) for each food. The software contained 1,832 items, registered after updating the database from the previous edition.

To estimate the amounts consumed in grams or milliliters of each food or beverage, we used the Table of Referenced Measures for Food Consumed in Brazil ( *Tabela de Medidas Referidas para os Alimentos Consumidos no Brasil* ), developed in POF 2008–2009 and reviewed and updated in POF 2017–2018^13^. Hereafter, we considered the quantities consumed on the first day of collection from the FR in 2008–2009 and the R24h in 2017–2018. Weekdays and weekends were represented in both surveys.

The reference person in the household answered a questionnaire with socioeconomic and demographic data, with information on age, education level, sex of the residents, and family income.

### Definition of Food Groups

In this research, food items were initially classified into 116 groups, described in the 2017–2018^11^ survey documentation. For different reasons, nine groups were excluded from the analysis: water, distilled beverages, wine, beer, supplements, mixed preparations, sauces and condiments, sweeteners, and sugars. We excluded alcoholic beverages because they were related to lifestyle and not necessarily to an eating pattern or behavior; supplements for not being considered food or drink; mixed preparations, sauces and condiments and sweeteners due to the low frequency of consumption (less than 5% of the population); and sugars (honey/ *rapadur* a and table sugar) because their consumption data were collected differently in the POF editions. For some beverages, especially fruit juices, added sugar was not calculated separately in POF 2008–2009, which could hinder comparisons. Finally, the 107 groups were regrouped according to eating habits and/or similarities in nutritional composition, resulting in 19 groups, described with their main constituent foods in Box 1 .

**Box 1:** Food groups and items. National Dietary Surveys 2008–2009 and 2017–2018.

Food groups	Food items
Rice	Rice, brown rice, rice-based preparations, corn and corn-based preparations and other cereals.
Beans	Beans, green beans/rope, bean-based preparations, meat substitutes and other legumes.
Leafy vegetables	Lettuce, kale, cabbage, raw salad and other leafy vegetables.
Vegetables	Pumpkin, carrots, chayote, cucumber, tomatoes and other vegetables.
Roots and tubers	Sweet potatoes, English potatoes, cassava and other tubers.
Fruits	Pineapple, *açaí* , banana, orange, apple, papaya, mango, watermelon, tangerine, grape, dried fruit, other fruits and oilseeds.
Flour and pasta	Cassava flour, *farofa* , pasta, instant noodles, noodles and noodle-based preparations.
Breads	Salt bread (bread roll, water bread, hamburger bread, wheat bread, salt bread, unspecified bread, unspecified white bread, *cacetinho, careca, ciabatta, bisnaguinha, Croissant* , industrialized loaf bread, of corn, toast, sweet toast, brioche, Arabic, for *wrap* , gluten-free, potato, homemade, bread with butter and bread with margarine) and wholemeal salt bread (Australian, rye, black, whole, whole Arabic, whole *cacetinho* , whole *careca* , wholerye, whole salt, whole wheat, whole roll, rye toast and whole toast).
Cakes and cookies	Breakfast cereals, baked sweets, cakes, stuffed cakes, sweet biscuits, savory biscuits, stuffed biscuits, snacks, chips and breads, cakes and biscuits diet/light.
Meat	Beef, beef preparations, pork, poultry, poultry preparations, fresh fish, preserved fish, salted fish, other fish, fish preparations, salted meat, other types of meat, sausage, sausage, mortadella, ham, other cold cuts and offal.
Eggs	Eggs and egg-based preparations.
Dairy products	Whole milk, skimmed milk, milk-based preparations, vitamins, cheeses, yoghurts, other dairy products and dairy products diet/light.
Sweets	Chocolates, chocolates powder, milk-based sweets, peanut-based sweets, fruit-based sweets, ice cream/popsicles, other sweets and sweets diet/light.
Oils and fats	Oils and fats (coconut milk, mayonnaise – sauce –, bacon, olives, butter with or without salt, bottle butter, margarine with or without salt, bacon, olive oil, soy oil, corn oil, coconut oil, crackling, pork lard, beef lard, palm oil and unspecified oil) and oils and fats *light* (mayonnaise - sauce - light, margarine light, butter with or without salt light *and* coconut milk light).
Beverages	Juices, soft drinks/industrialized juices, soft drinks/industrialized juices diet/light, dairy drinks, soy-based drinks and other non-alcoholic beverages.
Sodas	Soda and diet/light soda.
Coffee and tea	Coffee and tea.
Pizzas and snacks	Pizzas, fried and baked snacks, sandwiches and savory pies.
Soups and broths	Soups and broths.

### Statistical Analysis

Dietary patterns were obtained by factor analysis (FA), extraction by principal component analysis, followed by Varimax rotation^14^. Since the data come from a survey with a complex sampling design, FA was performed to the correlation matrix among the 19 food groups (in grams) and estimated considering the sampling complexity^15^.

To determine the number of factors for data representation, we considered eigenvalues greater than 1.5. None food group was excluded to keep the same groups in both periods, even those with little contribution (low communalities)^14^. Therefore, the groups of leafy vegetables, roots and tubers, flour and pasta, vegetables, eggs, and beverages, despite not having a communality greater than 0.1, were included in the calculation of factor scores.

Food items whose factor loading were greater than 0.30 in module were considered to name the factors. The patterns naming found was based on interpretability, on the characteristics of the food groups retained in each pattern, and on the terminologies already recognized in publications on dietary patterns in Brazil and other countries.

Finally, factor scores were computed, estimates of each individual’s location in the FA factors^14^. These values have a mean of zero and, the more positive, they mean greater adherence to the dietary pattern; the more negatives, the less adherence. Comparison of mean of scores by different characteristics of the population clarify whether there is a relationship between these characteristics and the patterns.

The factor scores obtained in the 2017–2018 survey were evaluated according to the classes of variables: sex, age, region, per capita income, and education level, using their means and 95% confidence intervals. Age was stratified into two categories: 19 to 39 years and 40 to 59 years. Education level was stratified into less than 9 years of study (incomplete primary education) and 9 or more years of study (completed primary education). To calculate the per capita income, the total monthly income, the total number of residents per consumption unit and the minimum wage in force on the reference date of the survey, January 15, 2018, were considered. Income ranges were defined as: up to 0.5 minimum wage; between 0.5 and 1 minimum wage; between 1 and 2 minimum wages; and greater than 2 minimum wages.

All analyzes were performed using the *SAS OnDemand for Academics* statistical package (SAS Institute Inc., Cary, NC, USA), considering the complexity of the sample design.

## RESULTS

We identified three dietary patterns, together explaining 32% of the total variance of the data in both surveys. The first dietary pattern, called “traditional”, was composed of food groups that are usual in the Brazilian diet: rice, beans, and meat. The factor loadings observed were similar between the two surveys, with a slight downward trend for rice and beans and an increase for meat in the 2017–2018 survey compared to the 2008–2009 survey. In 2017–2018, a negative factor loading was also observed for the fruit and dairy groups.

“Breads and butter/margarine” is the second pattern and was characterized by the food groups breads and oils and fats (including margarine/butter). In both surveys, this pattern had a negative factor loading for the cakes and cookies group. In 2008–2009, we identified a positive factor loading for the coffee and teas group, and a negative one for beverages and pizzas and snacks. Breads and oils and fats had higher factor loadings in 2017–2018.

We named the third pattern “western”, a diet characterized by sodas and pizzas, and snacks. This pattern showed negative factor loadings for the fruit group in both surveys, with lower factor loadings in the second survey for sodas and pizzas and snacks. In addition, in 2017–2018, flours and pasta, and sweets groups had positive factor loadings, while vegetables and eggs had negative factor loadings ( Table 1 ).

**Table 1:** Factor loadings, communalities (h2), eigenvalues and percentage of cumulative variance explained obtained by factor analysis in food consumption data for Brazilian adults, National Dietary Surveys 2008–2009 and 2017–2018.

Food groups	Traditional		Breads and butter/margarine		Western		h^2^	
			
	2008–2009	2017–2018	2008–2009	2017–2018	2008–2009	2017–2018	2008–2009	2017–2018
Rice	0,83	0,80	0,14	-0,04	-0,14	-0,28	0,73	0,73
Beans	0,80	0,78	0,18	0,03	-0,12	-0,21	0,68	0,65
Leafy vegetables	0,07	0,03	-0,06	0,14	-0,13	-0,25	0,02	0,08
Vegetables	0,19	-0,02	-0,05	0,02	-0,20	-0,32	0,08	0,10
Roots and tubers	0,15	-0,11	-0,11	-0,20	-0,02	-0,18	0,03	0,08
Fruits	-0,13	-0,31	-0,20	-0,23	-0,38	-0,39	0,20	0,30
Flour and pasta	-0,20	-0,16	-0,12	0,03	0,11	0,45	0,06	0,23
Breads	-0,21	0,02	0,78	0,89	0,06	-0,09	0,66	0,80
Cakes and cookies	-0,09	-0,06	-0,30	-0,41	-0,04	0,18	0,10	0,20
Meat	0,59	0,64	-0,10	-0,10	-0,02	-0,04	0,36	0,42
Eggs	0,18	-0,21	0,25	-0,08	0,00	-0,31	0,09	0,15
Dairy products	-0,24	-0,37	-0,23	-0,24	-0,24	-0,09	0,17	0,21
Sweets	-0,17	-0,08	-0,29	-0,08	0,10	0,33	0,13	0,12
Oils and fats	-0,18	-0,03	0,71	0,87	0,00	-0,07	0,54	0,76
Beverages	-0,01	0,11	-0,32	-0,22	-0,19	-0,02	0,14	0,06
Sodas	-0,01	0,08	-0,15	0,01	0,84	0,73	0,72	0,54
Coffee and tea	-0,03	0,15	0,61	0,20	-0,07	-0,21	0,38	0,11
Pizzas and snacks	-0,16	-0,08	-0,33	-0,16	0,69	0,59	0,61	0,38
Soups and broths	-0,48	-0,42	0,01	0,04	-0,24	-0,12	0,29	0,19
Eigenvalues	2,25	2,21	2,15	2,02	1,60	1,88		
Cumulative variance (%)	12	12	23	22	32	32		

The average age among adults was 37 years in 2008–2009 and 38.5 in 2017–2018. In 2008–2009, 49.8% of adults were female and in 2017–2018, 50.1%. In addition, in both surveys, most of the adult population was resident in the Southeast region (43.6% in 2008–2009 and 42.8% in 2017–2018) and had a per capita income greater than two minimum wages (32.7% in 2008–2009 and 37.3% in 2017–2018); they aged between 19 and 39 years old (57.9% in 2008–2009 and 53.3% in 2017–2018). Regarding education, in 2008–2009, 50% of individuals reported incomplete primary education, while in 2017–2018 this percentage was 30.3%.

Greater adherence to the “traditional” pattern was observed among men, residents of the Midwest region, and people with incomplete primary education. Less adherence was observed among women, aged between 40 and 59 years, residents in the North region and people with per capita income greater than 2 minimum wages compared to people with income between 0.5 and 2 minimum wages ( Table 2 ).

**Table 2:** Means of factor scores according to sociodemographic variables, National Dietary Survey 2017–2018.

Variables	Traditional			Breads and butter/margarine			Western		
		
	Mean	IC95%^a^		Mean	IC95%^a^		Mean	IC95%^a^	
Sex									
Female	-0,35	-0,38	-0,33	0,01	-0,01	0,03	-0,04	-0,07	-0,02
Male	0,28	0,25	0,32	0,08	0,06	0,11	0,16	0,13	0,18
Age group									
19–39	0,03	0,00	0,06	0,02	-0,01	0,04	0,17	0,14	0,20
40–59	-0,11	-0,14	-0,08	0,08	0,06	0,11	-0,08	-0,10	-0,05
Regions									
North	-0,20	-0,25	-0,14	0,08	0,02	0,14	0,05	0,01	0,09
Northeast	0,03	-0,01	0,06	-0,03	-0,06	0,00	0,01	-0,02	0,04
Southeast	-0,09	-0,13	-0,05	0,12	0,08	0,15	0,04	0,00	0,08
South	-0,02	-0,08	0,04	0,07	0,02	0,12	0,24	0,18	0,29
Midwest	0,19	0,11	0,27	-0,11	-0,15	-0,06	-0,05	-0,09	0,00
Per capita income^b^									
< 0,5 MW	-0,03	-0,10	0,03	0,07	0,02	0,12	-0,16	-0,20	-0,12
0,5–1 MW	0,04	-0,01	0,09	0,07	0,04	0,11	-0,04	-0,08	0,00
1–2 MW	0,00	-0,04	0,04	0,09	0,05	0,12	0,06	0,03	0,10
> 2 MW	-0,11	-0,15	-0,07	0,00	-0,04	0,03	0,16	0,12	0,20
Education level^c^									
Incomplete PE	0,08	0,04	0,11	0,04	0,02	0,07	-0,13	-0,15	-0,10
Full PE or more	-0,08	-0,11	-0,06	0,05	0,03	0,07	0,13	0,11	0,16

MW: minimum wage.

^a^ Confidence interval (CI) of 95% for the mean of the factorial score.

^b^ Per capita income categories were defined according to the per capita income of consumption units and the minimum wage in force on the reference date of the survey (R$954.00).

^c^ Incomplete primary education (PE) was defined as less than nine completed years of study.

Men, people aged between 40 and 59 years, residents of the Southeast region showed greater adherence to the “bread and butter/margarine” pattern, compared to those in the Northeast and Midwest, and people with income between 1 and 2 minimum wages per capita, compared to those with an income above 2 minimum wages. Residents of the Midwest region showed less adherence to this pattern. For the “western” pattern, greater adherence was observed among men, aged between 19 and 39 years, residents of the South region, individuals with per capita income greater than 2 minimum wages and those with education level equal to or greater than the primary education. Older women, people with an income of less than 0.5 minimum wage per capita and those with incomplete primary education showed less adherence to this pattern ( Table 2 ).

## DISCUSSION

Based on the 2008–2009 and 2017–2018 National Dietary Survey, we identified three patterns in the adult Brazilian population: “traditional”, “breads and butter/margarine” and “western”. The first pattern is characterized by rice, beans and meat; the second for breads and oils and fats (including margarine and butter); and the third for sodas, pizzas, snacks, and negative factor loadings for fruits. It was found that adherence to the identified patterns varied according to sex, age group, region, per capita income, and education level.

The rice and beans pattern, with the participation of meat, usually called “traditional”, is frequently observed in national studies with data on individual consumption^2^. However, the effects of adherence to this dietary pattern are controversial. Protective effects for BMI were observed by Sichieri^7^, in Rio de Janeiro, for both men and women, and by Cunha et al.^3^, also in Rio de Janeiro, who reported an inverse association with BMI and waist circumference in women. However, Vilela et al.^16^, in the Midwest region, reported a direct association between the traditional pattern and central adiposity. Selem et al.^17^, in a cross-sectional population-based study, found that participants with hypertension adhered more to the traditional pattern.

The consumption of rice and beans is advantageous in several nutritional aspects: good quality protein, a source of iron, folate, and fiber. However, meat consumption, particularly red meat, has been associated with an increased risk of diabetes, cardiovascular disease, and certain types of cancer^18^. It is a current and great the debate regarding healthy and sustainable diets simultaneously, where the consumption of plant foods predominates and the participation of red meat is reduced. The high consumption of meat is related to greater pressure on the limits of the planet, whether due to the emission of greenhouse gases or the use of land for pasture and production of grains for animal feed^19^. Despite the positive aspect of the presence of rice and beans in the “traditional” pattern, the excessive consumption of meat among the Brazilian population^11 , 20^ is a possible explanation for the controversial results.

In the second dietary pattern, the characteristic food groups are breads and oils, and fats, including margarine and butter. In 2008–2009, the participation of the “coffee and teas” group was verified and presented; in 2017–2018 it presented lower factor loading. In the INA 2017–2018, bread and oils, and fats groups were among the foods with the highest consumption frequencies. In 2008–2009, bread was mentioned by 63% of respondents, while oils and fats counted for 37.8%; 2017–2018 reported 50.9% and 46.8%, respectively^10 , 11^.

Coffee was the most frequently mentioned food in 2017–2018 (78.1% of the population) and in 2008–2009 (79%), which also refers to a very common habit, which is the consumption of bread with butter/margarine at breakfast and snacks. Baltar et al.^21^, analyzing breakfast in Brazilian adults in the INA 2008–2009, reported an inversely associated BMI in a diet composed by sausages, milk, cheese, coffee/tea, and bread.

In the third pattern, “western”, two groups stand out in both periods: pizzas and snacks, and sodas. These foods are characteristic of fast meals and mark a less healthy diet, commonly related to a Westernized consumption pattern, described in the literature as associated with a higher risk of chronic diseases (obesity, diabetes, cardiovascular diseases, and asthma)^22^. This pattern is also characterized by negative loadings for fruits and vegetables.

Both INA editions highlighted the absence of a dietary pattern characterized by fruits, leafy vegetables, and vegetables – a finding that can be explained by the low consumption of these items by the adult population in Brazil^11^. Unfortunately, fruits and vegetables do not participate with positive factor loadings in any of the patterns identified in this study. In one of the patterns (“western”), they present negative loadings higher than the cutoff point defined to characterize a significant contribution to the pattern. The consumption of these items is associated with better quality diets and a better epidemiological profile.

A meta-analysis that included Brazil evaluated *a posteriori* dietary patterns and central obesity, observing an association between healthy patterns and lower risk of central obesity^23^. Another meta-analysis^24^ investigating *a posteriori* dietary patterns concluded that greater adherence to a dietary pattern characterized by high intake of fruits, vegetables, complex carbohydrates and low intake of refined carbohydrates, processed meat, and fried foods is a positive strategy concerning type 2 diabetes.

We did not observe a pattern that could be considered healthy. Observational and clinical studies evidence that diets based on plant foods, rich in fruits, vegetables, and whole grains are associated with the prevention of chronic diseases. Plant foods are characterized by being rich in fiber and antioxidant nutrients, as well as by low energy density and low levels of saturated fat, nitrate, nitrite, and iron. These characteristics are associated with maintenance of body weight, increased glycemic control, improved lipid profile, reduced blood pressure, improved vascular health, reduced inflammation, and a better profile of the intestinal microbiota^25^.

Diet is the main modifiable determinant of most chronic diseases. It is essential to examine the possible contribution of dietary patterns to the occurrence of these diseases^1^. However, few studies investigate the determinants of dietary patterns in population studies. In the present study, sociodemographic factors exerted influence, corroborating Romeiro et al.^6^, who found greater adherence to the “traditional” pattern in individuals with less education level in the *Pró-Saúde* study ( *Estudo Pró-Saúde* ), and data from the Surveillance System for Risk and Protective Factors for Chronic Diseases by Telephone Survey ( *Vigitel* ) between 2007 and 2012^8^.

Adherence to the “traditional” pattern according to sex is inconsistent in the literature: Arruda et al. reported greater adherence among men, as in our study,^26^ in a cohort of young adults in São Paulo, but Romeiro et al.^6^ found reduced adherence among men. Jaime et al.^27^, found that regular consumption of beans was higher among men, individuals with no education, and those with incomplete primary education, analyzing food consumption data that are markers of healthy eating from the 2013 National Health Survey ( *Pesquisa Nacional de Saúde* ). However, the populations sampled in these studies differ from those in the present study. In Pró-Saúde^6^, the sample was the permanent civil servants from a public university in the state of Rio de Janeiro, and Arruda et al.^26^ reported data from a cohort in São Paulo. Our study has a population-based sample, representative of Brazil, with methods for evaluating individual consumption with good accuracy, which may explain the differences found.

As in previous studies^27^, the results here presented confirm differences in consumption and eating habits according to the five regions of the country. The “traditional” pattern had less adherence in the North region, and the “western” pattern had greater adherence among residents of the South region.

The socioeconomic level has an important impact on the life situation of individuals, determining access to services, goods, and products, including food. Medina et al.^30^found a better food consumption profile in social groups with higher income and higher education. Santos and Conde ^8^ found greater adherence to the “prudent” pattern among more educated individuals, and greater adherence to the “western” and “traditional” patterns among less-educated individuals. In the present study, individuals with higher income had greater adherence to the “western” pattern, and those with lower income had less adherence to this pattern.

This study has possible limitations, such as the use of factor analysis in the derivation of dietary patterns, a method that involves some arbitrary decisions, such as the grouping of food items, retention of factors, and naming. However, the dietary patterns identified were comparable with other studies. Another limitation is the change in dietary data collection methods between the two surveys analyzed, and the greater number of food items in the database in 2017–2018. As positive points, we can mention the use of nationally representative data and the consideration of sample complexity to derive the patterns.

## CONCLUSION

We observed the maintenance of dietary patterns in the Brazilian adult population analyzing data in 2008–2009 and 2017–2018 National Dietary Surveys. It was possible to identify three patterns: the first characterized by the consumption of rice, beans, and meat; a second characterized by the consumption of breads, and oils and fats; and a third characterized by sodas, pizzas, and snacks. None pattern presented an important participation of fruits, vegetables, and leafy vegetables. Adherence to these dietary patterns varied according to sociodemographic characteristics and housing macro-region.
