# Supplementary material for: Dietary patterns of Brazilian adults in 2008–2009 and 2017–2018
Source: Rev Saude Publica. 2021 Nov 12;55(Suppl 1):8s. doi: 10.11606/s1518-8787.2021055003437 (PMC8647980; doi:10.11606/s1518-8787.2021055003437)
Supplement: Translation

# Translation: Padrões alimentares de adultos brasileiros em 2008–2009 e 2017–2018

**Authors:** Anna Beatriz Souza Antunes, Diana Barbosa Cunha, Valéria Troncoso Baltar, Josiane Steluti, Rosangela Alves Pereira, Edna Massae Yokoo, Rosely Sichieri, Dirce Maria Marchioni

**Affiliations:** I Brasil Universidade do Estado do Rio de Janeiro. Instituto de Medicina Social. Programa de Pós-Graduação em Saúde Coletiva. Rio de Janeiro, RJ, Brasil; II Brasil Universidade do Estado do Rio de Janeiro. Instituto de Medicina Social. Departamento de Epidemiologia. Rio de Janeiro, RJ, Brasil; III Brasil Universidade Federal Fluminense. Instituto de Saúde Coletiva. Departamento de Epidemiologia e Bioestatística. Niterói, RJ, Brasil; IV Brasil Universidade Federal de São Paulo. Instituto de Saúde e Sociedade. Departamento de Políticas Públicas e Saúde Coletiva. Santos, SP, Brasil; V Brasil Universidade Federal do Rio de Janeiro. Instituto de Nutrição Josué de Castro. Departamento de Nutrição Social e Aplicada. Rio de Janeiro, RJ, Brasil; VI Brasil Universidade de São Paulo. Faculdade de Saúde Pública. Departamento de Nutrição. São Paulo, SP, Brasil

**Keywords:** Adulto, Comportamento Alimentar, Análise Fatorial, Fatores Socioeconômicos, Inquéritos sobre Dietas

## Abstract

**OBJETIVOS:**

Identificar padrões alimentares entre adultos brasileiros a partir dos Inquéritos Nacionais de Alimentação (INA) 2008–2009 e 2017–2018, verificando, nesse último período, a aderência aos padrões de acordo com fatores sociodemográficos e regiões brasileiras.

**MÉTODOS:**

Foram analisados dados do primeiro de dois dias de consumo alimentar de adultos (19–59 anos de idade) entrevistados nos INA 2008–2009 (n = 21.630) e 2017–2018 (n = 28.901). Os padrões alimentares foram derivados por análise fatorial exploratória a partir de 19 grupos de alimentos, considerando a complexidade do desenho amostral. Para o INA 2017–2018, os escores fatoriais foram avaliados de acordo com sexo, faixa etária, região, renda per capita e escolaridade.

**RESULTADOS:**

Foram identificados três padrões nos dois inquéritos: (1) “tradicional”, caracterizado por arroz, feijão e carnes; (2) “pães e manteiga/margarina”, caracterizado por pães, óleos e gorduras (incluindo margarina/manteiga) e, em 2008–2009, café e chás; e (3) “ocidental”, caracterizado por refrigerantes e pizzas e salgados, além de farinhas e massas e doces em 2017–2018. O padrão “tradicional” teve maior aderência entre homens, moradores da região Centro-Oeste e indivíduos com ensino fundamental incompleto. Para o padrão “pães e manteiga/margarina”, observou-se maior aderência entre o sexo masculino, indivíduos com idade entre 40 e 59 anos, da região Sudeste e com renda entre 1 e 2 salários-mínimos per capita. Indivíduos do sexo masculino, com idades entre 19 e 39 anos, da região Sul, com renda per capita maior que dois salários-mínimos e escolaridade igual ou maior que o ensino fundamental foram os que apresentaram maior adesão ao padrão “ocidental”.

**CONCLUSÃO:**

Os padrões alimentares identificados em 2008–2009 e 2017–2018 foram similares, com manutenção do padrão “tradicional”, que inclui arroz, feijão e carnes. A adesão aos padrões varia de acordo com sexo, faixa etária, região, renda per capita e escolaridade.

## INTRODUÇÃO

O papel da dieta na promoção da saúde e prevenção de doenças é difícil de elucidar, devido à complexidade da interação entre alimentos e nutrientes. Além da ingestão total de energia, a composição dietética é provavelmente o fator discriminador mais importante intra e entre as populações. Ela se reflete nos padrões alimentares, e por isso a análise dessa composição tem ganhado popularidade^1^.

No Brasil, os padrões alimentares foram investigados em diversos estudos nas últimas décadas, ficando evidente, de forma geral, a combinação característica da dieta brasileira, baseada em arroz e feijão, um padrão comumente denominado “tradicional”^2^. A análise de padrões alimentares, diferentemente da análise isolada de alimentos e nutrientes, considera as relações entre os diferentes itens alimentares consumidos, abrangendo a complexidade das combinações^1^. As estimativas de padrões alimentares, ao detectarem práticas cotidianas, subsidiam a formulação de diretrizes dietéticas, guias alimentares, programas e políticas públicas.

Entretanto, dados sobre a evolução dos padrões alimentares no país ainda são escassos^8^, apesar das alterações na dieta brasileira ao longo dos anos. Dentre essas alterações, destacam-se o importante aumento da participação dos ultraprocessados, a redução do consumo de feijão e o aumento do consumo de frutas e hortaliças (ainda inadequado)^9^.

O Inquérito Nacional de Alimentação (INA), conduzido em uma subamostra da Pesquisa de Orçamentos Familiares (POF) desde a edição de 2008–2009, permite obter estimativas do consumo alimentar individual para toda a população brasileira^10 , 11^. Dessa forma, o objetivo do presente estudo é identificar padrões alimentares entre adultos brasileiros a partir de dados dos INA de 2008–2009 e 2017–2018, verificando, no segundo período, a aderência aos padrões de acordo com fatores sociodemográficos e regiões brasileiras.

## MÉTODOS

### População de Estudo

O estudo foi realizado com a população participante das edições de 2008–2009 e 2017–2018 do INA, módulo da POF, pesquisa de abrangência nacional que tem como unidade de investigação o domicílio e é realizada por amostragem. A POF coleta informações sobre despesas familiares, condições de vida e hábitos de consumo das famílias brasileiras. Detalhes sobre o processo de amostragem e da definição da amostra mestra podem ser obtidos nas publicações oficiais do Instituto Brasileiro de Geografia e Estatística (IBGE)^10 , 11^.

Para o INA 2008–2009, os dados de consumo alimentar pessoal foram coletados para todos os moradores com idade superior ou igual a 10 anos de idade residentes em 13.569 domicílios selecionados aleatoriamente. Esses domicílios correspondem a uma subamostra de 24,3% dos 55.970 domicílios investigados na POF, totalizando informações de 34.003 moradores. Para a edição de 2017–2018, foram coletadas as informações de consumo alimentar individual para a mesma faixa etária (≥ 10 anos), em 20.112 domicílios selecionados aleatoriamente, correspondentes a uma subamostra de 34,7% dos 57.920 domicílios investigados na POF, totalizando informações de 46.164 moradores.

A presente análise inclui moradores adultos (de 19 a 59 anos), de ambos os sexos. Foram excluídas mulheres que eram gestantes e lactantes no período de coleta (1.111 em 2008–2009 e 1.249 em 2017–2018). A amostra final perfez um total de 21.630 pessoas em 2008–2009, e 28.901 em 2017–2018.

### Consumo Alimentar Individual

Os dados de consumo alimentar individual foram obtidos no questionário de consumo alimentar pessoal (bloco POF7) em ambas as edições das pesquisas estudadas. O instrumento utilizado para coletar as informações sobre alimentos e bebidas consumidos no domicílio ou fora dele foi diferente nas duas edições.

No INA 2008–2009, cada morador registrou em dois dias não consecutivos o seu consumo individual. Para realizar o registro alimentar (RA), os indivíduos foram orientados a anotar e relatar detalhadamente o nome dos alimentos consumidos, o tipo de preparação, a medida usada, a quantidade consumida e o horário e o local do consumo (no domicílio ou fora). Os registros eram revisados junto com os moradores por um agente de pesquisa, que no próprio domicílio passava as informações para um computador portátil, utilizando programa de entrada de dados criado para esse fim. O *software* continha uma base de dados de alimentos e bebidas de aproximadamente 1.500 itens cadastrados.

No INA 2017–2018, o consumo alimentar foi avaliado por meio de dois recordatórios alimentares de 24 horas (R24h), no domicílio do entrevistado, em dias não consecutivos. A entrevista foi desenvolvida seguindo um roteiro estruturado em estágios sequenciais, embasado no Método de Múltiplas Passagens^12^. Como na edição anterior, os agentes de pesquisa também utilizaram um programa de entrada de dados, instalado em um *tablet* . Para cada alimento, o programa disponibilizava a informação da quantidade consumida (unidade de medida e quantidade). O *software* continha 1.832 itens, cadastrados após a atualização do banco de dados da edição anterior.

Para estimar as quantidades consumidas em gramas ou mililitros de cada alimento ou bebida, utilizou-se a Tabela de Medidas Referidas para os Alimentos Consumidos no Brasil, desenvolvida na POF 2008–2009 e revisada e atualizada na POF 2017–2018^13^. Para essa estimativa, foram consideradas apenas as quantidades consumidas no primeiro dia de coleta do RA em 2008–2009 e do R24h em 2017–2018. Em ambos os inquéritos, houve representação dos dias de semana e final de semana.

A pessoa de referência do domicílio respondeu questionário com dados socioeconômicos e demográficos, com informações sobre idade, escolaridade, sexo dos moradores e renda familiar.

### Definição dos Grupos Alimentares

Na presente pesquisa, os itens alimentares foram inicialmente classificados em 116 grupos, descritos na documentação do inquérito 2017–2018^11^. Em seguida, por diferentes razões, nove grupos foram excluídos das análises: água, bebidas destiladas, vinho, cerveja, suplementos, preparações mistas, molhos e condimentos, adoçantes e açúcares. Excluíram-se as bebidas alcoólicas por estarem relacionadas ao estilo de vida, e não necessariamente a um padrão ou comportamento alimentar; os suplementos por não serem considerados alimento ou bebida; as preparações mistas, molhos e condimentos e adoçantes devido à baixa frequência de consumo (menos que 5% da população); e os açúcares (mel/rapadura e açúcar de mesa) em razão de seus dados de consumo terem sido coletados de forma diferente nas edições da POF. Para algumas bebidas, em especial sucos de frutas, o açúcar de adição não foi calculado separadamente na POF 2008–2009, o que poderia prejudicar a comparação dos padrões. Ao final, os 107 grupos foram reagrupados de acordo com hábitos alimentares e/ou por similaridades na composição nutricional, resultando em 19 grupos, descritos com seus principais alimentos constituintes no Quadro 1 .

**Quadro 1:** Grupos e itens alimentares dos Inquéritos Nacionais de Alimentação 2008–2009 e 2017–2018.

Grupos alimentares	Itens alimentares
Arroz	Arroz, arroz integral, preparações à base de arroz, milho e preparações à base de milho e outros cereais.
Feijão	Feijão, feijão verde/corda, preparações à base de feijão, substitutos da carne e outras leguminosas.
Verduras	Alface, couve, repolho, salada crua e outras verduras.
Legumes	Abóbora, cenoura, chuchu, pepino, tomate e outros legumes.
Raízes e tubérculos	Batata-doce, batata-inglesa, mandioca e outros tubérculos.
Frutas	Abacaxi, açaí, banana, laranja, maçã, mamão, manga, melancia, tangerina, uva, frutas secas, outras frutas e oleaginosas.
Farinhas e massas	Farinha de mandioca, farofa, massas, macarrão instantâneo, macarrão e preparações à base de macarrão.
Pães	Pão de sal (pão-francês, pão d’água, de hambúrguer, de sal, de trigo, pão branco não especificado, pão não especificado, pãozinho, cacetinho, careca, *ciabatta* , bisnaguinha, *croissant* , de forma industrializado, de milho, torrada, torrada doce, brioche, árabe, para *wrap* , sem glúten, de batata, caseiro, pão com manteiga e pão com margarina) e pão de sal integral (australiano, de centeio, preto, integral, árabe integral, cacetinho integral, careca integral, de centeio integral, de sal integral, de trigo integral, francês integral, pãozinho integral, torrada de centeio e torrada integral).
Bolos e biscoitos	Cereais matinais, doces panificados, bolos, bolos recheados, biscoito doce, biscoito salgado, biscoito recheado, salgadinhos chips e pães, bolos e biscoitos *diet* / *light* .
Carnes	Carne bovina, preparações à base de carne bovina, carne suína, aves, preparações à base de aves, peixes frescos, peixes em conserva, peixes salgados, outros pescados, preparações à base de pescados, carnes salgadas, outros tipos de carne, linguiça, salsicha, mortadela, presunto, outros frios e embutidos e vísceras.
Ovos	Ovos e preparações à base de ovos.
Laticínios	Leite integral, leite desnatado, preparações à base de leite, vitaminas, queijos, iogurtes, outros laticínios e laticínios *diet* / *light* .
Doces	Chocolates, achocolatados, doces à base de leite, doces à base de amendoim, doces à base de fruta, sorvete/picolé, outros doces e doces *diet* / *light*
Óleos e gorduras	Óleos e gorduras (leite de coco, maionese – molho–, toucinho, azeitona, manteiga com ou sem sal, manteiga de garrafa, margarina com ou sem sal, bacon, azeite de oliva, óleo de soja, óleo de milho, óleo de coco, torresmo, banha suína, banha bovina, óleo de dendê e óleo não especificado) e óleos e gorduras *light* (maionese – molho – *light* , margarina *light* , manteiga com ou sem sal *light* e leite de coco *light* ).
Bebidas	Sucos, refrescos/sucos industrializados, refrescos/sucos industrializados *diet* / *light* , bebidas lácteas, bebidas à base de soja e outras bebidas não alcoólicas.
Refrigerantes	Refrigerantes e refrigerantes *diet* / *light* .
Café e chá	Café e chá.
Pizzas e salgados	Pizzas, salgados fritos e assados, sanduíches e tortas salgadas.
Sopas e caldos	Sopas e caldos.

### Análise Estatística

Os padrões alimentares foram obtidos por análise fatorial (AF), com extração por análise de componentes principais, seguida por rotação Varimax^14^. Uma vez que os dados são oriundos de um inquérito com delineamento amostral complexo, a AF foi realizada em matriz de correlação com os 19 grupos alimentares (em gramas) e estimada considerando essa complexidade amostral^15^.

Para determinar o número de fatores para representação dos dados foram considerados autovalores ( *eigenvalues* ) maiores que 1,5. Optou-se por não excluir nenhum grupo alimentar com o intuito de manter os mesmos grupos nos dois períodos, mesmo aqueles com pouca contribuição (comunalidades baixas)^14^. Assim, os grupos verduras, raízes e tubérculos, farinhas e massas, legumes, ovos e bebidas, apesar de não apresentarem comunalidade superior a 0,1, foram incluídos no cômputo dos escores fatoriais.

Para nomear os fatores, foram considerados os itens alimentares cuja carga fatorial ( *factor loading* ) foi superior a 0,30, em módulo. A nomeação dos padrões encontrados se baseou na interpretabilidade, nas características dos grupos retidos em cada padrão e nas terminologias já reconhecidas em publicações sobre padrões alimentares no Brasil e em outros países.

Por fim, foram computados os escores fatoriais, estimativas de localização de cada indivíduo nos fatores da AF^14^. Esses valores têm média zero e, quanto mais positivos, significam maior adesão ao padrão; quanto mais negativos, menor adesão. Dessa forma, ao se comparar as médias desses escores para diferentes características da população, é possível verificar se há relação entre tais características e os padrões.

Os escores fatoriais obtidos no inquérito 2017–2018 foram avaliados de acordo com as classes das variáveis: sexo, idade, região, renda per capita e escolaridade, através das médias e intervalos de 95% de confiança. Para as análises, a idade foi estratificada em duas categorias: 19 a 39 anos e 40 a 59 anos. A escolaridade foi estratificada em menos do que 9 anos de estudo (ensino fundamental incompleto) e 9 ou mais anos de estudo (ensino fundamental completo). Para calcular a renda per capita, considerou-se a renda mensal total, o total de moradores por unidade de consumo e o salário-mínimo vigente na data de referência do inquérito, 15 de janeiro de 2018. As faixas de renda foram definidas em: até 0,5 salário-mínimo; entre 0,5 e 1 salário-mínimo; entre 1 e 2 salários-mínimos; e maior do que 2 salários-mínimos.

Todas as análises foram realizadas no pacote estatístico *SAS OnDemand for Academics* (SAS Institute Inc., Cary, NC, EUA), considerando a complexidade do desenho amostral.

## RESULTADOS

Foram identificados três padrões alimentares que, juntos, explicaram 32% da variância total dos dados em ambos os inquéritos. O primeiro padrão alimentar, denominado “tradicional”, foi composto por grupos de alimentos usuais da dieta do brasileiro: arroz, feijão e carnes. As cargas fatoriais observadas foram semelhantes entre os dois inquéritos, com ligeira tendência de redução para arroz e feijão e aumento para carnes no inquérito de 2017–2018 em relação ao de 2008–2009. Em 2017–2018 foi também observada carga fatorial negativa para os grupos frutas e laticínios.

O segundo padrão, denominado “pães e manteiga/margarina”, foi caracterizado pelos grupos alimentares pães e óleos e gorduras (incluindo margarina/manteiga). Em ambos os inquéritos, esse padrão apresentou carga fatorial negativa para o grupo bolos e biscoitos. Em 2008–2009, observou-se carga fatorial positiva para o grupo café e chás, e negativa para bebidas e pizzas e salgados. Os itens pães e óleos e gorduras apresentaram maiores cargas fatoriais em 2017–2018.

O terceiro padrão, denominado “ocidental”, foi caracterizado pelos grupos refrigerantes e pizzas e salgados. Esse padrão apresentou carga fatorial negativa para o grupo frutas em ambos os inquéritos, com menores cargas fatoriais no segundo inquérito para refrigerantes e pizzas e salgados. Além disso, em 2017–2018, os grupos farinhas e massas e doces apresentaram carga fatorial positiva, enquanto legumes e ovos apresentaram carga fatorial negativa ( Tabela 1 ).

**Tabela 1:** Cargas fatoriais, comunalidades (h2), autovalores e percentual da variância explicada acumulada obtidos por análise fatorial em dados de consumo alimentar de adultos brasileiros, Inquérito Nacional de Alimentação 2008–2009 e 2017–2018.

Grupos alimentares	Tradicional		Pães e manteiga/margarina		Ocidental		h^2^	
			
	2008–2009	2017–2018	2008–2009	2017–2018	2008–2009	2017–2018	2008–2009	2017–2018
Arroz	0,83	0,80	0,14	-0,04	-0,14	-0,28	0,73	0,73
Feijão	0,80	0,78	0,18	0,03	-0,12	-0,21	0,68	0,65
Verduras	0,07	0,03	-0,06	0,14	-0,13	-0,25	0,02	0,08
Legumes	0,19	-0,02	-0,05	0,02	-0,20	-0,32	0,08	0,10
Raízes e tubérculos	0,15	-0,11	-0,11	-0,20	-0,02	-0,18	0,03	0,08
Frutas	-0,13	-0,31	-0,20	-0,23	-0,38	-0,39	0,20	0,30
Farinhas e massas	-0,20	-0,16	-0,12	0,03	0,11	0,45	0,06	0,23
Pães	-0,21	0,02	0,78	0,89	0,06	-0,09	0,66	0,80
Bolos e biscoitos	-0,09	-0,06	-0,30	-0,41	-0,04	0,18	0,10	0,20
Carnes	0,59	0,64	-0,10	-0,10	-0,02	-0,04	0,36	0,42
Ovos	0,18	-0,21	0,25	-0,08	0,00	-0,31	0,09	0,15
Laticínios	-0,24	-0,37	-0,23	-0,24	-0,24	-0,09	0,17	0,21
Doces	-0,17	-0,08	-0,29	-0,08	0,10	0,33	0,13	0,12
Óleos e gorduras	-0,18	-0,03	0,71	0,87	0,00	-0,07	0,54	0,76
Bebidas	-0,01	0,11	-0,32	-0,22	-0,19	-0,02	0,14	0,06
Refrigerantes	-0,01	0,08	-0,15	0,01	0,84	0,73	0,72	0,54
Café e chá	-0,03	0,15	0,61	0,20	-0,07	-0,21	0,38	0,11
Pizzas e salgados	-0,16	-0,08	-0,33	-0,16	0,69	0,59	0,61	0,38
Sopas e caldos	-0,48	-0,42	0,01	0,04	-0,24	-0,12	0,29	0,19
Autovalores	2,25	2,21	2,15	2,02	1,60	1,88		
Variância acumulada (%)	12	12	23	22	32	32		

A idade média entre os adultos foi de 37 anos em 2008–2009 e de 38,5 em 2017–2018. Em 2008–2009, 49,8% dos adultos eram do sexo feminino, e em 2017–2018, 50,1%. Além disso, em ambos os inquéritos, entre os adultos, a maior parte da população era residente da região Sudeste (43,6% em 2008–2009 e 42,8% em 2017–2018), com renda per capita maior do que dois salários-mínimos (32,7% em 2008–2009 e 37,3% em 2017–2018) e idade entre 19 e 39 anos (57,9% em 2008–2009 e 53,3% em 2017–2018). Em relação à escolaridade, em 2008–2009, 50% dos indivíduos relataram ter ensino fundamental incompleto, enquanto em 2017–2018 esse percentual foi de 30,3%.

Observou-se maior aderência ao padrão “tradicional” entre os homens, os residentes da região Centro-Oeste e as pessoas com ensino fundamental incompleto. Menor aderência foi observada entre as mulheres, com idade entre 40 e 59 anos, os residentes da região Norte e as pessoas com renda per capita maior do que 2 salários-mínimos em comparação às pessoas com renda entre 0,5 e 2 salários-mínimos ( Tabela 2 ).

**Tabela 2:** Médias dos escores fatoriais de acordo com variáveis sociodemográficas, Inquérito Nacional de Alimentação 2017–2018.

Variáveis	Tradicional			Pães e manteiga/margarina			Ocidental		
		
	Média	IC95%^a^		Média	IC95%^a^		Média	IC95%^a^	
Sexo									
Feminino	-0,35	-0,38	-0,33	0,01	-0,01	0,03	-0,04	-0,07	-0,02
Masculino	0,28	0,25	0,32	0,08	0,06	0,11	0,16	0,13	0,18
Faixa etária									
19–39	0,03	0,00	0,06	0,02	-0,01	0,04	0,17	0,14	0,20
40–59	-0,11	-0,14	-0,08	0,08	0,06	0,11	-0,08	-0,10	-0,05
Regiões									
Norte	-0,20	-0,25	-0,14	0,08	0,02	0,14	0,05	0,01	0,09
Nordeste	0,03	-0,01	0,06	-0,03	-0,06	0,00	0,01	-0,02	0,04
Sudeste	-0,09	-0,13	-0,05	0,12	0,08	0,15	0,04	0,00	0,08
Sul	-0,02	-0,08	0,04	0,07	0,02	0,12	0,24	0,18	0,29
Centro-Oeste	0,19	0,11	0,27	-0,11	-0,15	-0,06	-0,05	-0,09	0,00
Renda per capita^b^									
< 0,5 SM	-0,03	-0,10	0,03	0,07	0,02	0,12	-0,16	-0,20	-0,12
0,5–1 SM	0,04	-0,01	0,09	0,07	0,04	0,11	-0,04	-0,08	0,00
1–2 SM	0,00	-0,04	0,04	0,09	0,05	0,12	0,06	0,03	0,10
> 2 SM	-0,11	-0,15	-0,07	0,00	-0,04	0,03	0,16	0,12	0,20
Escolaridade^c^									
EF incompleto	0,08	0,04	0,11	0,04	0,02	0,07	-0,13	-0,15	-0,10
EF completo ou mais	-0,08	-0,11	-0,06	0,05	0,03	0,07	0,13	0,11	0,16

SM: salário-mínimo.

^a^Intervalo de confiança (IC) de 95% para média do escore fatorial.

^b^Categorias de renda per capita foram definidas de acordo com o rendimento per capita das unidades de consumo e o salário-mínimo vigente na data de referência do inquérito (R$ 954,00).

^c^Ensino fundamental (EF) incompleto foi definido como menos de nove anos completos de estudo.

Apresentaram maior aderência ao padrão “pães e manteiga/margarina”: os homens, as pessoas com idade entre 40 e 59 anos, os residentes da região Sudeste, comparado àqueles das regiões Nordeste e Centro-Oeste, e as pessoas com renda entre 1 e 2 salários-mínimos per capita, em comparação àquelas com renda superior a 2 salários-mínimos. Os residentes da região Centro-Oeste apresentaram menor aderência a esse padrão. Para o padrão “Ocidental”, maior aderência foi observada entre os homens, com idade entre 19 e 39 anos, os residentes da região Sul, os indivíduos com renda per capita maior que 2 salários-mínimos e aqueles com escolaridade igual ou maior que o ensino fundamental. Apresentaram menor aderência a esse padrão as mulheres, com idade mais elevada, as pessoas com renda menor que 0,5 salário-mínimo per capita e aquelas com ensino fundamental incompleto ( Tabela 2 ).

## DISCUSSÃO

Foi possível identificar, com base nos Inquéritos Nacionais de Alimentação de 2008–2009 e 2017–2018, três padrões alimentares da população brasileira adulta que se mantiveram similares: “tradicional”, “pães e manteiga/margarina” e “ocidental”. O primeiro padrão é caracterizado por arroz, feijão e carnes; o segundo, por pães e óleos e gorduras (incluindo margarina e manteiga); e o terceiro por refrigerantes, pizzas, salgados e por apresentar carga fatorial negativa para frutas. Verificou-se que a adesão aos padrões identificados variou de acordo com sexo, faixa etária, região, renda per capita e escolaridade.

O padrão alimentar composto por arroz e feijão, com participação da carne, usualmente denominado “tradicional”, é observado com frequência nos estudos nacionais com dados de consumo individual^2^. Os efeitos da aderência a esse padrão, contudo, são controversos. Efeitos protetores para IMC foram observados por Sichieri^7^, no Rio de Janeiro, tanto para homens quanto para mulheres, e por Cunha et al.^3^, também no Rio de Janeiro, que relataram associação inversa com IMC e circunferência da cintura em mulheres. No entanto, Vilela et al.^16^, no Centro-Oeste, relataram associação direta entre o padrão tradicional e a adiposidade central. Selem et al.^17^, em estudo transversal de base populacional, verificaram que participantes com hipertensão aderiram mais ao padrão tradicional.

O consumo da dupla arroz e feijão é vantajoso em vários aspectos nutricionais: aporte de proteínas de boa qualidade, fonte de ferro, folato e fibra. No entanto, o consumo de carne, particularmente carne vermelha, tem sido associado a risco aumentado de diabetes, doença cardiovascular e certos tipos de câncer^18^. Além disso, atualmente é grande o debate sobre dietas saudáveis e simultaneamente sustentáveis, em que o consumo de alimentos de origem vegetal prepondere e a participação da carne vermelha seja reduzida. O consumo elevado de carnes está relacionado a maior pressão sobre os limites do planeta, seja devido à emissão de gases de efeito estufa ou ao uso de terra para pastagem e produção de grãos para alimentação animal^19^. Dessa forma, apesar do aspecto positivo da presença de arroz e feijão no padrão “tradicional”, o consumo de carnes, excessivo entre a população brasileira^11 , 20^, é uma possível explicação para os resultados controversos.

No segundo padrão, os grupos de alimentos característicos são os pães e óleos e gorduras, que incluem a margarina e a manteiga. Em 2008–2009 também foi verificada a participação do grupo “café e chás”, que esteve presente em 2017–2018 com cargas fatoriais mais baixas. No INA de 2017–2018, os grupos pão e óleos e gorduras estavam entre os alimentos com as maiores frequências de consumo. Em 2008–2009, o pão foi relatado por 63% dos pesquisados, enquanto os óleos e gorduras por 37,8%; em 2017–2018, por 50,9% e 46,8%, respectivamente^10 , 11^.

O café foi o alimento mais frequentemente citado em 2017–2018 (78,1% da população) e em 2008–2009 (79%), o que remete também a um hábito bastante comum, que é o consumo de pão com manteiga/margarina no desjejum e lanches. Baltar et al.^21^, analisando o desjejum em adultos brasileiros no INA 2008–2009, relatou que um padrão composto por embutidos, leite, queijo, café/chá e pão esteve inversamente associado ao IMC.

No terceiro padrão, “ocidental”, dois grupos se destacam em ambos os períodos: pizzas e salgados e refrigerantes. Esses alimentos são característicos de refeições rápidas e demarcam uma alimentação menos saudável, comumente relacionada a um padrão de consumo ocidentalizado, descrito na literatura como associado a maior risco de doenças crônicas (obesidade, diabetes, doenças cardiovasculares e asma)^22^. Esse padrão também se caracteriza por cargas negativas para frutas e vegetais.

Destaca-se, nas duas edições do INA, a ausência de um padrão alimentar caracterizado por frutas, verduras e legumes – achado que pode ser explicado pelo baixo consumo desses itens pela população adulta no Brasil^11^. É desafortunado que frutas, legumes e verduras não participem com cargas fatoriais positivas em nenhum dos padrões identificados neste estudo, e que apresentem, em um dos padrões (“ocidental”), valores negativos superiores ao ponto de corte definido para caracterizar significante contribuição ao padrão. O consumo desses itens tem sido associado a dietas de melhor qualidade e melhor perfil epidemiológico.

Metanálise que incluiu o Brasil avaliou padrões dietéticos a posteriori e obesidade central, observando associação entre padrões saudáveis e menor risco de obesidade central^23^. Outra metanálise^24^, que também investigou padrões a posteriori, concluiu que maior aderência a um padrão alimentar caracterizado por elevada ingestão de frutas, legumes e carboidratos complexos, e baixa ingestão de carboidratos refinados, carne processada e alimentos fritos, mostrou-se uma estratégia com influência positiva na carga global de diabetes tipo 2.

Em nosso estudo, não foi observado um padrão que pudesse ser considerado saudável. A partir de estudos observacionais e clínicos, têm se acumulado evidências de que dietas à base de alimentos vegetais, ricas em frutas, legumes e grãos integrais estão associadas à prevenção de doenças crônicas. Os alimentos de origem vegetal se caracterizam por serem ricos em fibras e nutrientes antioxidantes, bem como pela baixa densidade energética e baixos níveis de gordura saturada, nitrato, nitrito e ferro. Essas características estão associadas a manutenção do peso corporal, aumento do controle glicêmico, melhora do perfil lipídico, redução da pressão arterial, melhora da saúde vascular, diminuição da inflamação e melhor perfil da microbiota intestinal^25^.

A dieta é o principal determinante modificável da maioria das doenças crônicas, e é essencial examinar a possível contribuição do padrão alimentar para a ocorrência dessas doenças^1^. No entanto, são escassos os estudos que investigam os determinantes dos padrões alimentares em estudos populacionais. No presente estudo, verificou-se a influência de fatores sociodemográficos, corroborando Romeiro et al.^6^, que verificaram maior aderência ao padrão “tradicional” em indivíduos com menor escolaridade no estudo Pró-Saúde, e dados do Vigitel entre os anos de 2007 e 2012^8^.

A adesão ao padrão “tradicional” de acordo com gênero é inconsistente na literatura: maior aderência entre homens, como no nosso estudo, foi relatada por Arruda et al.^26^em uma coorte de adultos jovens em São Paulo, mas Romeiro et al.^6^verificaram adesão reduzida entre homens. Jaime et al.^27^, analisando dados de consumo de alimentos marcadores de alimentação saudável da Pesquisa Nacional de Saúde 2013, verificaram consumo regular de feijão maior entre homens, indivíduos sem instrução e com ensino fundamental incompleto. No entanto, as populações amostradas nesses estudos diferem das do presente estudo. No Pró-Saúde^6^, a amostra foi formada por servidores públicos efetivos de uma universidade pública do estado do Rio de Janeiro, e Arruda et al.^26^relataram dados de uma coorte em São Paulo. Nosso estudo tem amostra de base populacional, representativa do Brasil, com métodos de avaliação de consumo individual com boa acurácia, o que pode explicar as diferenças encontradas.

Como em estudos anteriores^27^, os resultados aqui apresentados confirmam diferenças de consumo e hábito alimentar de acordo com as cinco regiões do país. O padrão “tradicional” apresentou menor aderência na região Norte, e o padrão “ocidental” maior aderência entre residentes da região Sul.

O nível socioeconômico tem impacto importante sobre a situação de vida dos indivíduos, determinando o acesso a serviços, bens e produtos, entre eles alimentos. Medina et al.^30^verificaram melhor perfil de consumo alimentar nos grupos sociais de maior renda e maior escolaridade. Santos e Conde^8^verificaram maior aderência ao padrão “prudente” entre indivíduos mais escolarizados, e maior aderência aos padrões “ocidental” e “tradicional” entre indivíduos menos escolarizados. No presente estudo, os indivíduos com maior renda apresentaram maior adesão ao padrão “ocidental”, e aqueles com menor renda menor adesão a esse padrão.

Este estudo tem possíveis limitações, como o uso da análise fatorial na derivação dos padrões alimentares, método que envolve algumas decisões arbitrárias, como o agrupamento dos itens alimentares, a retenção dos fatores e sua nomeação. Contudo, os padrões alimentares identificados foram comparáveis com outros estudos. Outra limitação é a mudança de métodos de coleta de dados dietéticos entre os dois inquéritos analisados, e o maior número de itens alimentares na base de dados em 2017–2018. Como pontos fortes, pode-se mencionar a utilização de dados nacionalmente representativos e a consideração da complexidade amostral para derivar os padrões.

## CONCLUSÃO

Analisando os dados dos Inquéritos Nacionais de Alimentação de 2008–2009 e 2017–2018, observa-se manutenção de padrões alimentares da população adulta brasileira. Foi possível identificar três padrões: um primeiro caracterizado pelo consumo de arroz, feijão e carnes; um segundo caracterizado pelo consumo de pães e óleos e gorduras; e um terceiro caracterizado por refrigerantes, pizzas e salgados. Em nenhum padrão observou-se participação importante de frutas, legumes e verduras. Houve variação na adesão a esses padrões de acordo com características sociodemográficas e macrorregião de moradia.
